# The Ethical Issues of Social Media Companies and the Attention Economy Business Model. A Systematic Scoping Literature Review

**DOI:** 10.1007/s11948-026-00611-1

**Published:** 2026-07-27

**Authors:** Mahon McCann, Fiachra O’Brolchain, Joss Moorkens, Bert Gordijn

**Affiliations:** Institute of Ethics, Dublin City University, Dublin, Ireland

**Keywords:** Social media, Social media ethics, Attention economy, Digital platform regulation

## Introduction

This paper systematically reviews the literature on the ethics of social media companies (SMCs) with the attention economy business model (ABM). The term “social media” refers to a loose group of “Web 2.0” information technologies that emerged in the first decade of the 21st century (Vallor, 2022). e.g., Facebook, Instagram, YouTube, LinkedIn, Twitter, Snapchat, and TikTok. Despite becoming many of the largest and most successful companies in the world, these SMCs give away access to their platforms for free to users and instead profit from advertisers and third parties. In the literature, this business model (giving away the product for free and generating revenue from advertising) is referred to as the advertising or ABM. While the platforms are free for users to access, the advertisers pay the companies for these users’ data and the opportunity to influence the users’ behaviour to their preferences. Thus, the business model is rooted in user attention, because the data of users is extracted from their attentional habits and then sold to the advertisers and third parties for profit (Myllylahti, 2018).

The ABM began traditionally in television, newspapers and radio. However, the critical difference between these traditional models and the social media internet model is the real-time collection of user data; user data is described as the ‘lifeblood’ of the ABM (Ghosh & Scott, 2018, p. 1). The user data, through tracking attention on the platform, is used to build a predictive model of the user’s behaviour to better target ads and messages to increase the chances of advertisers’ success (Ghosh & Scott, 2018). Employing the ABM incentivises the platform to gather as much data as possible for advertisers and hence to get as many users as possible onto the platform^1^ and keep the users active on the platform for as long as possible. When the ABM is employed, the advertiser is the customer, and the user becomes the product. As former Facebook and Google Chief Engineer Justin Rosenstein put it, “If you are not paying for the product, you are the product” (McDavid, 2020).

The incentive structure created by the ABM is often discussed in the public conversation as leading to the use of addictive and manipulative design techniques like infinite scrolls, misaligned content-ranking algorithms and intermittent variable rewards by SMCs. This misaligned incentive structure came to public consciousness with the Cambridge Analytica scandal of 2016, the Facebook whistleblower Frances Haugen in 2021, the Wall Street Journal (2021) article “The Facebook Files”, and Tristan Harris’s documentary “The Social Dilemma” (McDavid, 2020). These scandals drew mainstream media attention to the ethical infractions of SMCs, such as third-party manipulation of individual behaviour, misinformation, privacy issues and threats to democracy. Based on the Digital Markets Act of 2022, the EU even required Meta to offer a genuine alternative to the data-driven advertising business model for users in 2024 and then consequentially fined Meta €200 million for failure to comply in 2025 (European Commission, 2025).

Despite these recent regulatory attempts to change the ABM, the misaligned incentive structure has permanently shaped the digital landscape. Ethan Zuckerman, inventor of the pop-up ad, described the ABM as the ‘original sin’ of the internet (Zuckerman, 2014). The early entrants to the market, like Facebook and LinkedIn, established this ‘zero-price’ paradigm and monetised through advertising, needing to gather as many free users as possible to make the ABM viable. The success of these companies, through employing the ABM, is an obstacle to newcomers to the market establishing themselves as viable competitors and so incentivises the use of the same addiction design techniques despite not employing the ABM. For example, Mastodon or Bluesky, which are mainly funded by donations, sponsorship or grants, still employ the same manipulative and addictive design techniques, like infinite scrolls or intermittent variable rewards. When the price is zero, then SMCs compete for a finite pool of attention instead, and this zero-price market was only made possible in the first place by employing the ABM.

The employment of the ABM by the largest and most successful SMCs has led to an attentional *tragedy of the commons*, whereby new entrants into the market must play the same attention game to succeed. If SMCs had begun with the platforms as a paid product, seeking to profit from users’ money rather than their attention, this tragedy would not have taken place. Arguably, the platforms might not have been successful either, but this tragedy of the commons dynamic highlights why dealing with the misaligned incentive structure of the ABM first, would have the largest effect on the social media landscape.

Despite the mounting evidence of the problematic consequences of SMC and attribution to the misaligned incentives of the ABM in the public sphere, no study has been published to review the academic literature on the ethical issues recorded in relation to SMCs’ ABM thus far. Against this background, we will systematically review the academic literature with a focus on 1) ethical issues identified and 2) solutions to the ethical issues proposed. First, we detail how sources were searched, selected and analysed. Then, we address each of the above-mentioned research aims in the results section. Finally, we offer a critical reflection on the results in the discussion section.

## Methodology

**Fig. 1:**
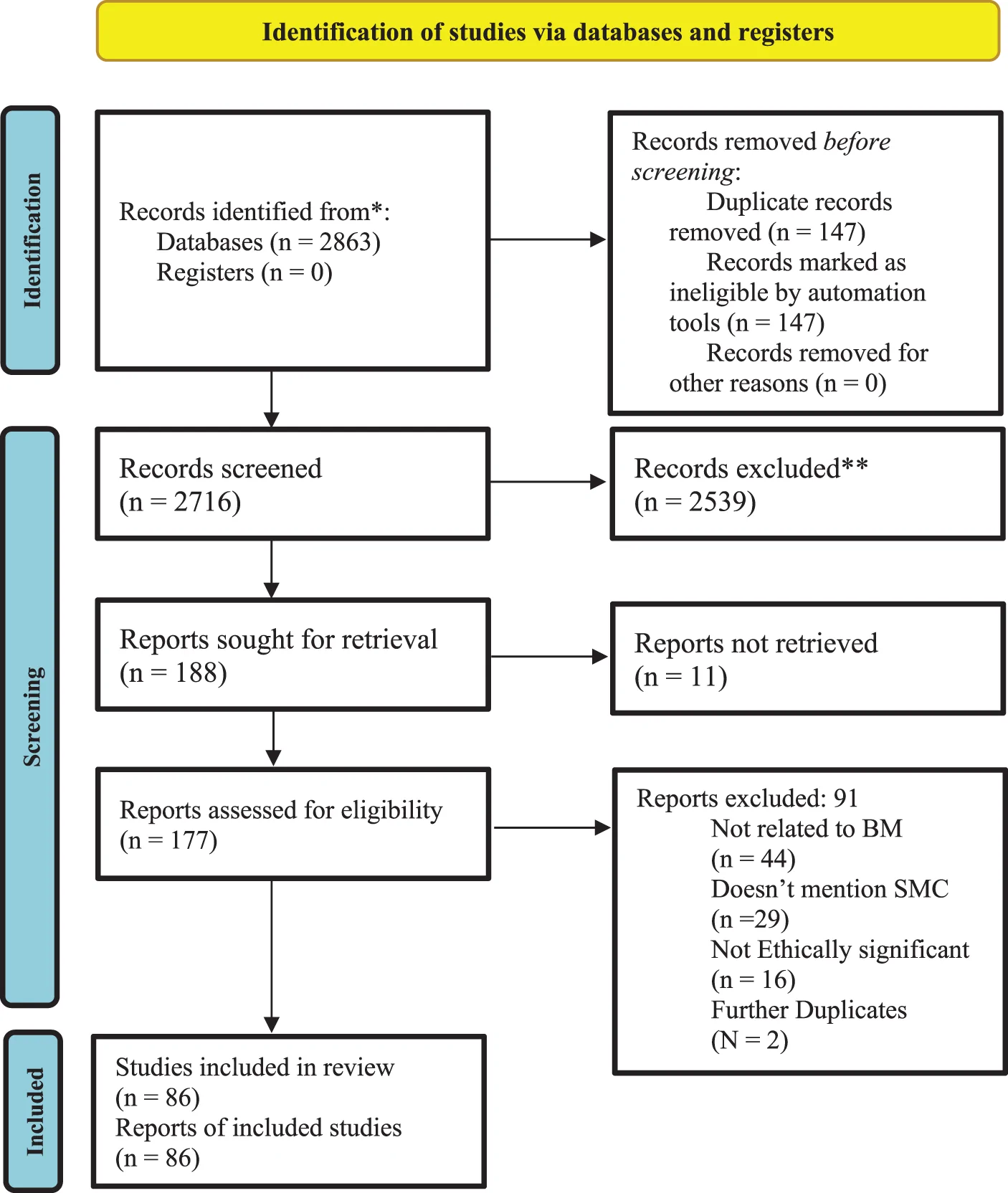
PRISMA flow diagram

We followed a systematic scoping method using the PRISMA guidelines for this literature review (as seen in Fig. 1). As PRISMA is generally a method for quantitative reviews, we have adapted the method qualitatively by focusing on identifying ethical issues, ethical arguments and solutions in the literature and will report according to the PRISMA-ScR checklist (Tricco et al., 2018). Since the social media literature spans several disciplinary fields, the following databases were searched: 1) ScienceDirect, 2) Scopus, 3) Google Scholar, 4) Web of Science 5) PsychInfo. Across these databases are publications of professional reports and societies, book chapters and grey literature, peer-reviewed papers and online resources, all covering a wide variety of research disciplines.

A comprehensive search strategy was developed in each database. The search strategy incorporated terms describing SMCs, including the main individual social networks (‘Facebook’ OR ‘Instagram’ OR ‘Snapchat’ OR ‘TikTok’ OR ‘YouTube’ OR ‘Twitter’), the terms ‘ethics’ OR ‘ethical issues’, keywords and available controlled vocabulary (MeSH) describing the business model of SMCs. Subsequently, we created three sets of search terms. Search set one initially included terms for the concept of ethics (e.g., ‘ethics’, ‘values’, ‘virtues’, ‘obligations’, and ‘morals’). However, including these search terms removed numerous relevant sources. Therefore, instead of a controlled vocabulary, it was decided to identify the ethical aspects during the screening process. The second set contained terms describing SMCs (e.g., ‘Social Media’ OR ‘Social Network*’ OR ‘Information Technology’ OR ‘Facebook’ OR ‘Instagram’ OR ‘Snapchat’ OR ‘TikTok’ OR ‘YouTube’ OR ‘Twitter’). The third set, joined with a Boolean AND, focused on the aspect of attention monetisation with ‘Attention Engagement’ OR ‘Attention Economy’ OR ‘Attention Monetisation’ OR ‘Attention Economy Business Model’ OR ‘Social Media Business Model’. We adopted the attention monetisation strategy (*see full search strings in the Appendix)* for all five databases (with modifications for ScienceDirect to fit 8 Boolean operators and Google Scholar to fit the 256-character restraint). The researchers searched Google Scholar for additional studies and reviewed the reference list of included studies for relevant citations.

The searches in the different databases resulted in 21 sources from Psychinfo, 251 in Science Direct, 98 in Web of Science, 123 in Scopus, and 2260 in Google Scholar. An additional 110 relevant citations were identified through a backward snowballing exercise. The total unique records identified after removing 147 duplicates were 2716. The records identified belong to four categories: 1) peer-reviewed journal articles, 2) books and book chapters, 3) commentaries, reviews, and editorials, and 4) conference workings and proceedings. To assess the eligibility of each record, we screened the titles, abstracts and keywords; this includes conference proceedings and workings. For books and book chapters, we screened the introduction and table of contents.

The eligibility criteria were twofold: inclusion and exclusion. To be included, a record must discuss to a significant degree the ethical issues of SMCs’ ABM, either explicitly or implicitly.^2^ Publications published before 2012 and non-English sources were excluded. The reason sources before 2012 were not considered is that the technology of social media developed rapidly in the early 2010s; for example, Facebook introduced the ‘like’ button, and so much of the early literature was outdated by this next generation of platforms. When these inclusion and exclusion criteria were applied to the 2716 records identified, they were assessed by the first author, examining titles, abstracts and keywords. This yielded 188 relevant results. The same author then conducted a second screening by reading these 188 records in full to evaluate their relevance to the study. Based on this full record reading and review with other authors, we excluded a further 91 based on the inclusion and exclusion criteria, which left 86 unique records to be included in the final review (11 results in full text were not retrieved online or via inter-library loans and so were excluded). This process is depicted in the PRISMA flowchart (Fig. 1), showing each stage of the selection process.

To open code the included sources in the review, a data-charting table was set up in Microsoft Excel to determine what information will be extracted from each source and included the following columns: Author(s), Title, Journal, Research Purpose, Research Question, Research Methodology, Ethical Issue(s) and Solutions. The data analysis and synthesis was performed according to inductive qualitative content analysis, which is common for reviews of normative information (Mertz et al., 2017, p. 10). As the sources were read in detail and reviewed, the researcher extracted ethical issues from the defined purpose or problem of each paper as reported by the paper’s authors. To prevent reporting bias, the first author charted the data, and in case of ambiguities or concerns about particular sources, feedback was sought from the co-authors. The data items charted included ethical issues and ethical solutions described in the included literature.

We are not defining ethical issues from the perspective of a specific ethical theory. Instead, we have chosen a pluralistic approach in identifying the ethical issues. The term ‘ethical issues’ is used to describe a violation of values, norms, consequences, rules, ethical principles or virtues broadly understood, either as reported by the authors themselves in the records or as identified by the authors of this literature review. This pluralistic method is chosen to identify ethical issues from different ethical perspectives such as consequentialism, deontology and virtue ethics and not to confine the identification of ethical issues to a particular ethical theory. For example, in Angwald et al. (2021), “Prediction accuracy and autonomy”, the problem discussed is how recommender systems try to predict the intentions of users from their behavioural data rather than from users’ expressed desires. The underlying normative value this violates is user *autonomy*. The threat to autonomy in this case is manipulation by recommender systems, and so the ethical issue of this paper would be classified as manipulation but under the ethical cluster of autonomy. See all ethical issues identified across the literature under the headings of the four ethical clusters in the review in Table 1 below.

**Table 1:** Ethical clusters & issues within those clusters

Ethical Clusters	Ethical Issues within the clusters			
*Autonomy*	Manipulation	Addiction	Distraction	
*Unfair Market Practice*	Privacy & Excessive Advertising	Attention Monopolies	Content Moderation vs Free Speech	Cybercrime/terrorism
*Democracy* *& The Public Sphere*	Misinformation/ disinformation	Content Moderation vs Free Speech	Polarisation	
*Platform Governance*	Self-Regulation	External- Governance	Third-party Governance	

**Fig. 2:**
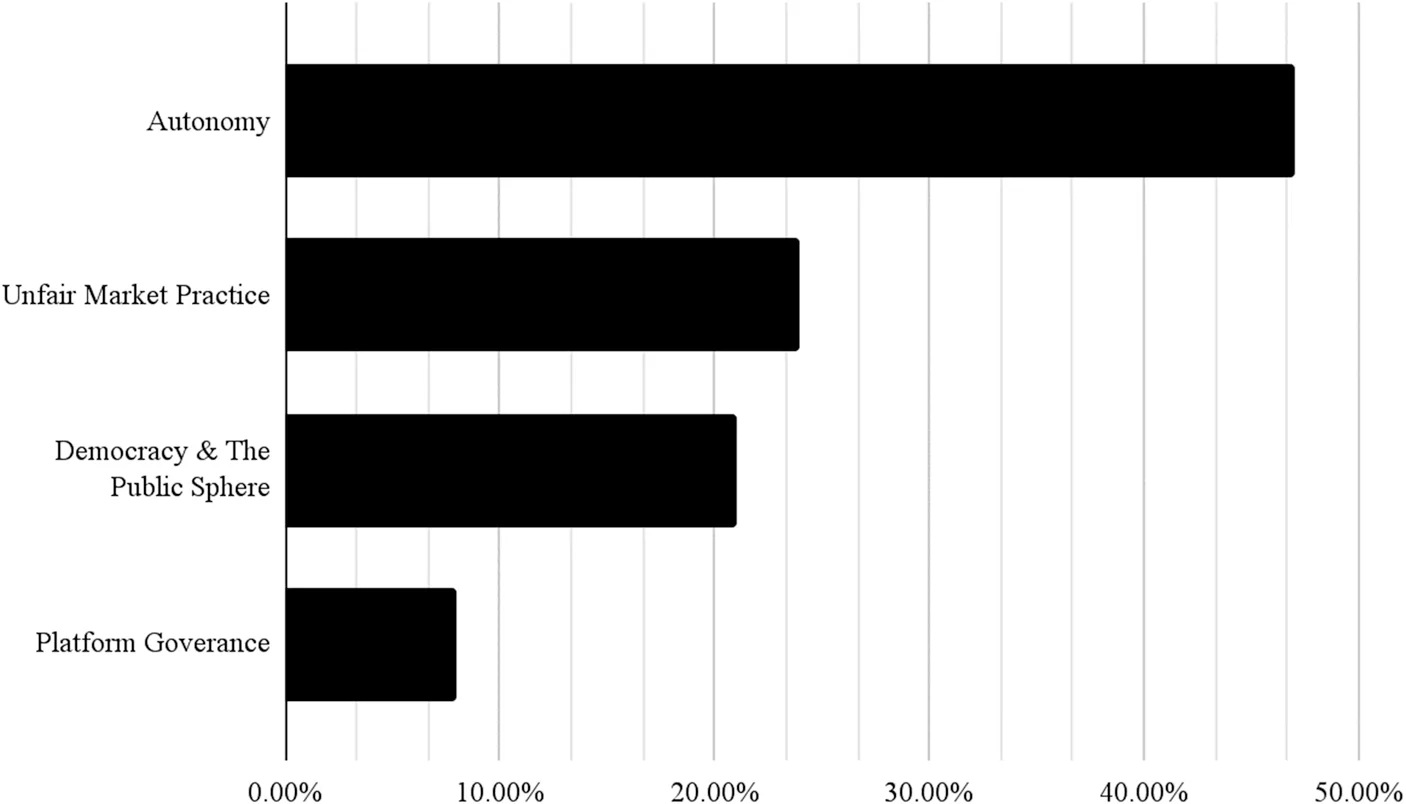
Ethical clusters

As the ethical issues were extracted and categorised from the included sources, overarching themes in the ethical issues emerged bottom-up. These broader themes were identified as four ethical clusters (autonomy, unfair market practice, democracy and the public sphere, and platform governance), and the ethical issues were categorised under these four broader ethical clusters to serve as a thematic basis for future research. This organisation of the material, as ethical issues within broader ethical clusters, is chosen because it reflects the organisation of issues across the interdisciplinary literature and reveals connections and commonalities within the issues, making them more amenable to ethical examination. The ethical clusters emerged from the observation that ethical issues generally fell across micro- (personal or individual harms) or macro-scales (economic, political, and social harms). The micro-scale issues are addiction, distraction and manipulation; issues like monopolies and governance are on the macro-scale, while the social issues of misinformation and disinformation or polarisation travel across both the macro and the micro levels. The ethical clusters chart the various ethical issues across the micro-scale and macro-scales, separating out the economic, political and social harms. In Fig. 2, the percentage of records examined in the review in each ethical cluster is reported.

The results are presented as a structured narrative organised by the emergent categories across the ethical issues and ethical clusters. We categorised and qualitatively explored extracted aspects based on their frequency of occurrence in the literature, exploring similarities and differences across the ethical issues. Since this review aims to provide a synthesis of published literature, we assumed equal weight for all ethical aspects discussed in a significant way. This is because attempting to rank ethical aspects at this point has theoretical and practical drawbacks, and it is beyond the scope of this review. We are limiting the critical appraisal of these sources in the review because (1) assigning more weight to an ethical aspect could imply that the moral principle(s) associated with that aspect has more weight than others and should be ranked higher and (2) assigning more weight to an ethical aspect could imply higher urgency or significance. It is reasonable to claim that the urgency or significance of ethical aspects might differ for each stakeholder (e.g., user versus policymaker), but exploring the perspectives of different stakeholders about SMCs is beyond the scope of this review.

## Results

### Autonomy

The most prominent ethical cluster in the literature is autonomy.^3^ Autonomy is dealt with by 47% of the papers (40 out of 86). We are grouping the ethical issues of 1) manipulation, 2) addiction, and 3) distraction under the heading of autonomy, because in the literature these ethical issues are considered to be violations of an individual’s autonomy or freedom.

#### Manipulation

The most reported ethical issue in the autonomy cluster is manipulation^4^ (21 out of 40 records). Manipulation is defined as influence by controlling the content and supply of information in such a way that a person is not aware of it (Williams, 2017, p.16). Manipulation occurs through hidden influences that interfere with people’s decision-making process to steer them toward the manipulator’s ends, exploiting people and influencing them in a non-transparent and covert manner (Kozyreva et al., 2020). Therefore, manipulation is considered unethical because it involves bypassing reasoning or some form of trickery or pressure (Fasoli, 2021).

The type of manipulation discussed is attentional manipulation, where SMCs shape attentional patterns and architecture outside our awareness (Gardiner, 2022, p. 14; Pedersen et al., 2021). Kärki (2022) separates three levels of this manipulation: affecting automatic attentional processes through nudging or immersing, diminishing the agent’s metacognitive capacity to control attention, and creating ideals and norms by making something ubiquitous in the attentional environment. Similarly, Sahebi and Formosa (2022) emphasise the social aspect of SMCs’ manipulation, for example, where the exhibition of wealth and material possessions influences perceptions of beauty, social status, and social capital within the social media world.

Several features of SMC platform design are implicated in this manipulation:

*Personalisation.* One of the core claims of manipulation in the literature is that social media sites’ collection of user data to personalise micro-targeted ads is manipulative. The literature discusses the idea of ‘data personas’ or ‘data shadows’, archived traces of user action that capture patterns of behaviour to make predictions which can facilitate the influencing of their decision-making process with timed communications (Paasonen, 2018). The way in which widespread collection of user data facilitates personalised micro-targeted ads is frequently considered to be manipulative (Karr & Craig, 2019; Lewandowsky et al., 2020). Multiple pieces of demographic data could be reliably inferred from people’s data personas, including ethnicity, occupation, and sexual orientation, and computer-based prediction of personality traits from digital personas is generally more reliable than human predictions (Kozyreva et al., 2020). This information can be employed to persuade and manipulate users; for example, advertising messages can be adjusted to match people’s introversion or extroversion score (Kozyreva et al., 2020). This constant surveillance and information tailoring can be used to manipulate users, and through a lack of transparency, control or choice, users cannot opt out of the personalisation system (Rufus, 2020).

*Dark Patterns.* Users are manipulated by dark patterns in platform design (Kidron et al., 2018; Schoenherr, 2022; Montalvo et al., 2021). One example of a dark pattern is YouTube’s autoplay feature, which removes “stopping friction”. Participants in a YouTube study described finding it difficult to resist engaging in “just one more video”, which could become a couple of hours of watching. This unplanned use can lead to regret and undermine a user’s sense of agency (Lukoff et al., 2021).

*Recommender systems.* In a related but distinct critique, due to the phenomenal amount of information uploaded to SMCs, filtering via algorithms is necessary to navigate. However, the goal of filtering is not user relevance but user engagement. The optimisation for user engagement optimises for the “short-term” self over the aspirational long-term self (Lukoff et al., 2021). In other words, the environment adapts to the user’s actions to keep them engaged (Marin, 2022). Lanier argues SMC algorithms are manipulative because they are adaptive, i.e., the algorithm makes changes in real-time to get better results: “As the algorithm tries to escape a rut, the brain becomes stuck in one” (Lanier, 2018, p. 15). This automation of recommender systems is trying to pre-empt the user’s agency so that the futures that are desirable to the company over the user are selected and other options undesirable to the company are foreclosed (Goodwin, 2022, p. 3).

*Gamification.* SMCs use gamification to encourage participation, such as Snapchat streaks, instant messaging, and rank-ordering posts. Gamification offers initial motivation to participate but aims to form habitual use through incentivising rewards. One of the examples of online gamification discussed in the literature is that Twitter introduces “game reasons” (Jongepier & Klenk, 2022, p. 7) into human communication through shares, likes and retweets; this is manipulation because people act then for game reasons rather than their own (Jongepier & Klenk, 2022).

Many records offer solutions to the problem of manipulation through data and advertising regulation and consumer education. Manipulation in SMCs is identified as being “data-driven” (Goodwin, 2022, p. 6). Without user data, personalised targeted advertisements, or ‘micro-targeting’, are not possible. Therefore, Paterson recommends reform to data privacy law, creating robust mechanisms for obtaining consent for data collection and use similar to GDPR (Paterson et al., 2021). Paterson also argues that a more robust response would be to ban micro-targeted advertising altogether, banning tracking and profiling techniques. The call to totally ban personalisation or micro-targeted advertising is prominent to reduce manipulation (Paterson et al., 2021; Jongepier & Klenk, 2022; Lanier, 2018; Lawand, 2021; Owen, 2019; Rufus, 2020; Torbert, 2021). Value-positive design is suggested as an alternate design paradigm **(**Steinert & Dennis, 2022; Spiekermann et al., 2022)

#### Addiction

The ethical issue of addiction is the use of persuasive design techniques to induce addiction in users and generate demand. A total of 20% of all the records examined dealt with addiction issues related to the ABM (17 out of 86).^5^ Most of the literature discusses addictive design features, or persuasive design techniques, that leverage individuals’ psychological biases to promote unhealthy use of SMC applications (Angwald et al., 2021; Aswad, 2020; Baughan et al., 2022; Beattie, 2022; Bhargava & Velasquez, 2021; Burr & Florida, 2020; Chianella, 2021; Fasoli, 2021; Hanin, 2021; Hartford & Stein, 2021; Kozyreva et al., 2020; Lanier, 2018; Mujica et al., 2022; Pillion et al., 2022; Sun & Zhang, 2021; Vanden Abeele et al., 2022). In the discussion of addictive design features, three of these design elements are shared across records:

Firstly, the ‘slot machine effect’, which is how SMCs leverage intermittent variable rewards (Williams, 2017). Several papers include that SMC platforms have been designed for variable rewards, i.e., notifications, messages, and novel stimuli, which encourage users to build a “checking habit” and engage the brain’s reward system similar to the anticipation of winning the lottery, gambling, or eating food (Baughan et al., 2022, p. 4). These variable rewards are built into design patterns such as infinite scrolling, autoplay and instant messaging and reinforce using behaviour by checking for variable rewards. Using variable rewards is habit-building and seems to create a constant state of alertness in users even when not using the applications (Mujica et al., 2022).

Second, design features take advantage of our desires for social validation and social reciprocity (Bhargava & Velasquez, 2021). This type of design builds a system of social rewards and punishments to encourage engagement (Lanier, 2018). These design features include gaining followers, getting likes and shares and keeping up a streak on platforms like Snapchat (Aswad, 2020). The social punishments include loss of status or reputation, social ostracisation, or simply being ignored. They take advantage of human cognitive biases such as the fear of missing out, social comparison, status quo bias, framing effects, and anchoring effects (Williams, 2017).

Thirdly, platform design erodes natural stopping cues. One example is the “infinite scroll” that prevents users from reaching a natural endpoint at the bottom of the page, which continuously loads content to continue engagement (Bhargava & Velasquez, 2021). This fosters normative dissociation (Baughan et al., 2022), or a “flow-state”, similarly to gambling technologies, which get users addicted by helping them to enter “the zone” or “the world-dissolving state of subjective suspension and affective calm they derive from machine play” (McKelvey & Hunt, 2019, p. 5).

The incentive to use persuasive design techniques comes from ABM’s relationship between user engagement, selling advertising and revenue (Mujica et al., 2022). The more time users spend engaged on the platforms, the more advertising revenue the companies make (Lanier, 2018; Bhargava & Velasquez, 2021). The long-term effects of social media addiction include increased depression, anhedonia, anxiety, and issues with stress, loneliness and insomnia (Bhargava & Velasquez, 2021; Mujica et al., 2022; Castro & Pham, 2020; Lanier, 2018).

Several records suggest design interventions to address the problems of addictive design.

Proposals range from an ethically orientated designer’s oath that imposes transparency norms for persuasive design tools (Williams, 2017) to the prevention of normative dissociation (Baughan et al., 2022) to customisable recommendations (Baughan et al., 2022; Lukoff et al., 2021; Mujica et al., 2022). Numerous records also recommend making the platforms as easy to leave as they are to sign up for (Aswad, 2020; Bhargava & Velasquez, 2021; Mujica et al., 2022), greater customisability (Kozyreva et al., 2020), education and self-management skills (Mujica et al., 2022), and banning social media technology for minors (Bhargava & Velasquez, 2021). A frequent recommendation is the banning/reform of the ABM (Bhargava & Velasquez, 2021; Hartford & Stein, 2021; Lanier, 2018; Mujica et al., 2022; Rosenquist et al., 2022). Kärki (2022) argues that the attention economy should be regulated as an addictive good, a product that centres on habit-forming and harm-inducing products. Most conclude that without dealing with the ABM, other interventions to curb addiction will be ineffective.

#### Distraction

Only two records exclusively deal with distraction^6^ (2 out of 40), which is mostly dealt with under the heading of addiction. The ethical issue at stake is that SMCs with the ABM are incentivised to habitually distract users, or what Atchley and Lane (2014) describe as our distraction-rich world. There is a different conceptual debate in the literature around the ethical issue of distraction versus addiction, which is a more extreme case. Hanin (2021) explores the social and technological changes that have led to a rise in digital distraction in the attention economy, such as increased and almost unavoidable smartphone ownership, persuasive design features, and the use of harvested behavioural data facilitating personalised targeting.

Distraction is discussed obliquely in many of the records on addiction and manipulation. Williams defines distraction as the direction of one’s attention from information that is salient to one’s authentic goals (Williams, 2017). He describes three categories of distraction: *functional*, *existential*, and *epistemic*. A functional distraction distracts from one’s immediate goals, e.g., getting a notification while trying to write an essay. Existential distractions direct someone’s attention away from their ultimate values or “being goals” (Williams, 2017, p. 82) to lower-level goals, i.e., scrolling Facebook to avoid an important decision about one’s future. Epistemic distraction undermines people’s ability to pursue or define goals. Williams’ implication of the business model connects threats to Autonomy and distractions as manipulation of the will, that frustrate the authorship of one’s life (Hartford & Stein, 2021)

The recommended solutions for the problem of digital distraction resemble those of addiction, centring around transparency around persuasive design techniques, norms of advertising and increased advertising regulation. Also recommended are the creation of new social norms around technology, educational strategies, parental oversight, and informational initiatives similar to anti-smoking and anti-drunk driving campaigns (Hanin, 2021). He also recommends government promotion of “technology-lite environments” whereby architecture and design help to reduce distractions (Hanin, 2021, p. 403) and suggests that “freedom of attention” is a human right (Hanin, 2021, p. 404).

### Unfair Market Practices

The second most discussed issue is unfair market practices,^7^ which are discussed in 24% of the records (21 out of 86). An unfair market practice deals with the ethical issues of SMCs’ monetisation of user attention. Social media companies compete in attention markets, alternatively ‘zero-price markets’, which refer to SMCs that provide goods and services free of monetary charge. The SMC operates in a two-sided market as a platform intermediary, bringing buyers and sellers together and facilitating their transactions like a credit card company, shopping mall, or eBay (Wu, 2015). The platform brings together the users and the advertisers and lowers the price on one side to zero, making its profit on the advertiser side (Wu, 2015).

Attention is the main currency for social media companies, and ‘time spent’ is considered much like spending money in a traditional market for goods and services (Parisi & Parisi, 2021). Therefore, the companies “compete for eyeballs” (Wu, 2015, p. 1), rather than money, as they would in a typical money market. The ethical issues in this ethical cluster are to do with violations of values, norms or ethical principles of free market practice by social media companies’ commodification or monetisation of attention as recorded in the literature. These ethical issues identified are 1) privacy and excessive advertising and 2) attentional monopolies.

#### Privacy & Excessive Advertising

Most of the literature on unfair market practices discusses the excesses of digital advertising and behavioural data collection^8^ for advertising personalisation. These excesses violate norms of market practice and advertising with regard to individual privacy (12 out of 21). The ethical issue is that all forms of customer engagement (Ormen & Gregersen, 2022) on social media platforms are recorded and monitored for personalisation of content and ads. Shosanna Zuboff called this behavioural data collection for advertising “surveillance capitalism” (Zuboff, 2019).

Some of the data generated by our actions on a platform is used to improve the platform for our benefit, but then the surplus is sold to other companies. The behavioural surplus can be called “surveillance assets”; this can be turned into “surveillance revenues” and translates into “surveillance capital” (Zuboff, 2019). Zuboff calls this new type of market power *‘instrumentarianism’* – ‘defined as the instrumentation and instrumentalisation of behaviour for the purposes of modification, prediction, monetisation and control’ (Zuboff, 2019, p. 357).

This business model raises a personalisation-privacy paradox because customers want and need some personalisation to navigate the internet. But how much privacy to sacrifice for personalisation is decided by the companies and not the users, therefore raising questions about user dignity and freedom (Cloarec, 2020).

Marketers can exploit customer data for personalisation (Cloarec, 2020), and the influence given by so much data to marketers can be used by third parties to encourage consumption (Kish, 2020). The point of advertising is to create desires in customers and hence influence the elasticity of demand for a product, increasing prices and consumption (Wu, 2015). These new methods of behavioural data collection led digital advertising from an estimated $37B market in 2007 to over $360B by 2020 (Baeza-Yates & Fayyad, 2022).

The externalities created for consumers from invasive digital advertising are addressed in the section on ‘autonomy’, but this type of behavioural advertising raises ethical questions around market manipulation as well as individual manipulation. Traditional television advertising is ‘probabilistic’. As one television advertiser noted, “Half the money I spend on advertising is wasted; the trouble is I do not know which half” (Linford, 2020, p. 129). However, the internet model is more *deterministic*, i.e., a platform with enough users and enough behavioural data can promise specific results over large enough statistical populations (Lanier, 2018). Some argue this deterministic element means the companies are no longer doing advertising, as advertising requires a limited window to pitch and the possibility of failure; dynamically optimised stimuli fed to a large enough population to modify behaviour do not have the same constraints (Lanier, 2018).

It is argued in a two-sided market that the payment taken from advertisers is the company’s revenue; therefore, the advertising charges are the ‘price’ of the product (Bostoen, 2019). The behavioural data collection of the SMC, unlike the traditional advertisers, therefore allows the platform to modify the environment (UI/UX) to generate more data for the advertisers and hence generate more revenue. This capacity to modify the medium in real time to capture more data and attention to profit from advertisers is truly novel in social media platforms and drives the excesses of digital advertising (Goodwin, 2022). Unlike traditional media, which innovates on the level of content, the social media platforms innovate on the level of the medium to capture more attention for advertisers, which is why the platforms are proving so difficult to regulate (Goodwin, 2022)

To address the issues of privacy and excessive digital advertising, Balkin recommends treating SMCs as information fiduciaries (creating duties of care, confidentiality, and loyalty). The point of applying this role is to change how SMCs think about their end users and their obligations to their end users, not as commodities but as individuals (Balkin, 2021). This change in attitude from the companies towards their users could lead to respecting privacy and reducing the negative externalities of excessive advertising. Wu recommends consumer protection legislation to protect consumers from this non-consensual attention capture and prevent uncompensated transfers of attentional resources, mainly when they are captive audiences (Wu, 2018). Many of these solutions provided can be boiled down as attempts to incentivise companies to prioritise individual privacy and well-being over their advertising revenues.

#### Attention Monopolies

The second most discussed issue in relation to unfair market practices is the anti-competition issue of attention monopolies^9^ (9 out of 21). Attention monopolies are an ethical issue because they block new entrants into the market that might challenge existing companies and compete on things like safety and user experience (Nelson, 2021) and that the monopolist essentially controls the means of media production (Wold, 2022).

The anti-competitive behaviour of monopolists creates stagnation in the market and prevents customers from availing of other options. This ‘bottle-neck power’ means customers might experience high switching costs, such as loss of valued data or reputational indicators at the point of switching (Mittelstadt & Laux, 2021). The monopolist might employ contract terms that deter switching and technical barriers such as complex processes or a lack of interoperability between services to discourage competition (Mittelstadt & Laux, 2021). Therefore, attentional monopolies limit customers’ choices and healthy competition in the social media market.

A frequent critique of anti-competitive behaviour from SMCs is that these monopolies occur through illegitimate mergers. Wu argues that Facebook’s acquisition of Instagram was anti-competitive because this was a big competitor in the attention market and that mergers under US and UK law should be queried if they lessen competition (Wu, 2018). Rosenquist et al. (2022) also argue that WhatsApp and Instagram were two of Facebook’s biggest competitors and so they should not have been able to merge. As a result of its serial acquisitions, Facebook now owns three of the top four social networks, measured by worldwide users (Rosenquist et al., 2022, p. 477). These mergers give the social media giants huge access to user data and hence predictive power for advertisers and so increase their share of the market to a problematic degree.

Similarly, Parisi and Parisi (2021) and Rahman (2018) argue that SMCs are natural monopolies because of their *network effects* (Metcalfe, 2013). Parisi and Parisi argue this is because of the network effects on the consumption side for their users; a network effect is when the product or service gains more value as more people use it (Parisi & Parisi, 2021). Software is relatively cheap to build versus traditional infrastructure like roads and railways; hence, what makes the platform valuable is the increase in users, which makes it more attractive to advertisers and other users (Parisi & Parisi, 2021). Rahman argues that the value of the service increases radically as more users engage in the platform, and therefore new platforms are less likely to be able to displace established platforms with similar products because of these network effects (Rahman, 2018).

There is some disagreement in the literature about social media monopolies, and Parisi and Parisi note that the FTC (Financial Trade Commission) did not consider Facebook a natural monopoly in their 2020 antitrust case. Others suggest that the ad-intermediary industry is heavily dominated by a few actors, suggesting an oligopoly (Mittelstadt & Laux, 2021) or a duopoly in the digital advertising space: Facebook and Google (Balkin, 2021). Parisi and Parisi actually see a monopoly as ultimately desirable in the attention market because it limits the attentional costs on users and charges higher prices to advertisers (Parisi & Parisi, 2021).

There are several suggestions for addressing the SMC monopoly. Parisi and Parisi examined “structural”, “behavioural”, and “market-based” remedies (Parisi & Parisi, 2021, pp. 7, 15, 22). A standard structural recommendation is the breaking up of large SMCs like Facebook in order to remove market concentration and increase competition and new entries into the market or prevent future mergers and takeovers that might hinder them. Behavioural antitrust remedies focus on remedying the anti-competitive behaviour of firms in concentrated markets, such as price regulation, price caps and controls, or in attention markets, “attention caps” and “attention taxes” (Parisi & Parisi, 2021, p. 17). As a market-based solution, this record recommends a Chadwick–Demsetz scheme, which uses competition over ownership of the monopoly as a substitute for competition within the market – bidders would compete on customer protection as well as price in a government-mandated auction (Parisi & Parisi, 2021). Parisi and Parisi ultimately argue that market-based remedies will be most effective. Balkin recommends using competition law to create competition, prevent early buy-ups and separate different functions housed in the same company (Balkin, 2021). Rahman (2018) and Rochefort (2020) argue that in the case of a natural monopoly, making the monopoly public infrastructure is warranted, and this would be a kind of structural regulation.

### Democracy & the Public Sphere

The third most reported ethical issues were identified in the cluster on Democracy and The Public Sphere,^10^ appearing in 21% of the records (18 out of 86). Social media platforms are in large part used for global communication. This ability to communicate globally and social media companies’ goals of maximising attention capture come with certain risks that are perceived to violate norms of democratic discourse and public civility in the literature. These violations create a number of ethical dilemmas around content moderation and freedom of speech, verifying the veracity of information online, fuelling political polarisation, and inciting violence. In the following sections we will look in-depth at each of the ethical issues that violate norms of communication and democracy identified in the literature. The ethical issues are 1) misinformation and disinformation, 2) content moderation and free speech, 3) polarisation and 4) cybercrime and terrorism.

#### Misinformation & Disinformation

The most reported ethical issue in this cluster is misinformation and disinformation, reported in 50% of the records examined in this section (9 out of 18). 11 Disinformation is defined in the literature as sharing *intentionally* misleading information, possibly for financial or political ends, while misinformation is defined as sharing false or misleading information without malicious intent (Duberry, 2022; Ryan et al., 2019; Shadmy, 2022).

Several records link misinformation and disinformation to the ABM (Balsillie, 2019; Brady et al., 2020; Buiten, 2022; Ghosh & Scott, 2018; Owen, 2019; Ryan et al., 2019; Shadmy, 2022). Several papers argue that while the platforms are against misinformation and disinformation, their financial incentives align with creators of fake news because a successful disinformation campaign drives engagement on the platform (Brady et al., 2020; Ghosh & Scott, 2018; Rathje et al., 2021; Shadmy, 2022). The main argument is that the incentives of the ABM, optimising for user engagement and attention maximisation, favour“entertainment, shock and radicalization over reliable information“(Owen, 2019, p. 3). Virality is often achieved through“high-arousal emotions” (Brady et al., 2020, p. 980; Rathje et al., 2021, p. 1), moral language and“out-group animosity” (Brady et al., 2020; Rathje et al., 2021, p. 2; Sussman, 2021). Brady et al. (2020) report a roughly 20% increase in sharing on average per tweet with each moral-emotional word added (Brady et al., 2020). The dilemma for SMCs is that reducing out-group animosity and incentives for emotional and moral language would reduce engagement and profit (Rathje et al., 2021).

The solutions to misinformation and disinformation generally start with increased transparency being a key focal point. Sussman argues U.S. regulators can and should demand *transparency* from tech companies on how their platforms function and legislators should have active teams of researchers and social scientists, funded by government and tech companies, to help develop regulation and safety in tech (Sussman, 2021). Others recommend a searchable digital advertising database for each platform with information about political ads (e.g., sponsor, spend level and targeting parameters) and transparency around data collection and persuasive design techniques to allow users more control and insight (Ghosh & Scott, 2018).

Government regulation to adapt content-ranking algorithms is suggested as an effective treatment to reduce disinformation campaigns (Brady et al., 2020; Shadmy, 2022). Buiten argues for giving platforms editorial responsibility, similar to broadcasters or publishers, for the platform’s user-generated content, therefore incentivising self-regulation of disinformation and misinformation (Buiten, 2022). Duberry recommends the use of AI to assist moderators in “fact-checking” and identification of disinformation campaigns (Duberry, 2022, p. 179). For users, the recommendations are “boosting”, which is training to enhance users’ cognitive and motivational capacities (Kozyreva et al., 2020, p. 105); programmes of education in digital literacy to boost the competencies of users (Ghosh & Scott, 2018; Kozyreva et al., 2020; Marin, 2022; Pennycook & Rand, 2021); and education (Pennycook & Rand, 2021).

#### Content Moderation & Free Speech

As the digital public square, any regulation of communication on social media has consequences for the epistemic environment, democratic discourse and an individual’s right to freedom of speech. This ethical issue accounts for 33% of the records examined in this section (6 out of the 18).^12^

SMCs play a significant role in determining the public conversation (Balkin, 2021; Collins et al., 2020; Scott, 2019) and have responsibilities to remove inappropriate and illegal user-generated content (Heldt & Dreyer, 2021). For the platforms, the balancing act is between preventing harm and preserving individual rights such as copyrights, democratic values, public safety, cognitive liberty, and personality rights (Heldt & Dreyer, 2021; Stjernfelt & Lauritzen, 2020). Content moderation at scale is difficult because of the number of moderators needed and the cost of human moderation; this is why SMCs are mostly exploring automated content moderation options (Balkin, 2021). This automated content moderation raises censorship issues (Kopf, 2022) and lacks judgement and sensitivity to context, which is why automated fact-checking struggles with conversational sources (Duberry, 2022, p. 179).

Few SMC issues have garnered as much attention as content moderation and freedom of speech or freedom of expression, with the diverse regulatory environment indicating how difficult it is to balance removing harmful content and avoiding excessive restrictions on freedom of expression (Buiten, 2022). Heldt & Dreyer note that SMCs are not as “content-neutral” as they claim, given that the content selected for is the most contentious (Heldt & Dreyer, 2021, p. 269). They recommend both bottom-up moderation mechanisms (e.g., creating features for user moderation such as upvotes and downvotes) and a third-party governance structure, which is independent of the company and would be held accountable to a bill of social norms, the public interest and the common good (Heldt & Dreyer, 2021). Others discuss designing platforms to achieve better public ends (Balkin, 2021) or the fostering of democratic discourse and better communication (Brady et al., 2020; Shadmy, 2022, p. 2).

#### Polarisation

This ethical issue is focused on whether or not social media communication fuels political polarisation. Polarisation is discussed exclusively in 11% of the records examined (2 out of 18) (Van Bavel et al., 2021; Hopster, 2021). However, it is also frequently mentioned in the other literature and is considered a significant ethical issue.

There is a debate in the broader literature about whether or not social media’s business model contributes to political polarisation. Hopster identifies platforms as driving users into “echo chambers” and incentivising inter-group conflict (reference), while some argue that social media has no role in political polarisation (Van Bavel et al., 2021, p. 913). Van Bavel argues that social media is not the primary driver of polarisation but is a “key facilitator”. Van Bavel identifies several key features that could contribute to political polarisation: 1) Partisan Selection, 2) Message Content and 3) Platform Design and Algorithms. Similarly, Hopster argues that social media offers affordances to populism, which he defines in terms of political polarisation and splitting into a political ‘in-group’ and ‘out-group’ (Hopster, 2021).

Partisan selection is based on human cognitive bias, such as confirmation bias, that incentivises individuals to seek information confirming their held position. Van Bavel argues that there is research to support that individuals seek out confirming information and only update their beliefs when that information supports what they already believe and that they are more likely to share politically congruent information online (Van Bavel et al., 2021). Message content we have discussed previously in the misinformation and disinformation section, but Van Bavel argues that divisive content gets more engagement, is more likely to go viral, and hence incentivises ‘out-group animosity’ and the use of emotional-moral language (Van Bavel et al., 2021). Hopster echoes this point in identifying that social media algorithms allow sensationalist claims to spread comparatively easily and that populists can take advantage of ‘crisis’ messaging (Hopster, 2021). Finally, polarisation seems to be platform dependent. For instance, researchers in Israel found more polarisation on Twitter than on Facebook or WhatsApp, and some platforms seem to promote content that reinforces one’s social identity and pre-existing beliefs (Van Bavel et al., 2021).

In conclusion, Van Bavel identifies the limits of connecting political polarisation and social media platforms; that polarisation might drive social media use instead of the other way around; and the ultimate limitations of transparency because of researchers’ lack of access to social media platforms’ inner workings (Van Bavel et al., 2021). Hopster, however, focuses on the affordances of social media that fuel populism and that it is the populism which fuels political polarisation, providing an alternate hypothesis (Hopster, 2021). Whilst polarisation is an issue that cannot be completely solved by regulatory intervention, many design features that fuel polarisation online are built into the platforms and the business model. At the very least, the recommendations are further research and regulation for polarisation on social media (Van Bavel et al., 2021) or actual algorithmic design and policy changes to improve social media conversations (Rathje et al., 2021).

#### Cybercrime & Terrorism

The ethical issues of cybercrime and cyberterrorism are focused on social media companies’ roles in promoting violence in the social sphere. They were discussed exclusively in 1 record examined out of 18 (Rauf, 2021), but they also featured in others and are a significant topic.

The main topic of discussion was social media’s role in acts of violence, racism and terrorism (Rauf, 2021). Rauf links social media to terrorist acts through three steps: *subculturisation, spectacularisation and popularisation.* The first describes how individuals can find extremist communities online, then, using the example of the Christchurch shooting in New Zealand, how social media can spectacularise acts of violence in real time (live-streaming the attack, for example). After the fact, the content is then popularised to other potential extremists (Rauf, 2021).

These are similar to mechanisms described in the previous sections that promote unethical content throughout social media because of the aim of attention maximisation, and Rauf explicitly links the incentives for such violence and terror to the incentives of the attention economy, providing the latest coverage of events, spreading information, and algorithmic amplification (Rauf, 2021). Rauf argues that attention given to the acts of terror encourages further fear and extremism, and Rauf links this directly to the current economics of attention (Rauf, 2021).

In terms of solutions, it is noted that SMCs are not entirely responsible for cybercrime and terrorism on the platforms. However, Rauf (2021) argues that SMCs need to consider the ethical design facets of products, utilising stricter ethical protocols in testing and hiring ethical specialists. He suggests that the companies need to hire specific stress-testing teams who work on manual editing of the platforms and can disengage features instantly, e.g., live streaming, to minimise spectacularisation (Rauf, 2021). Undertaking these preventative measures could ensure less violence in the future.

### Platform Governance

The fourth ethical cluster examined is platform governance,^13^ which accounted for 8% of the records examined (7 out of 86). The ethical cluster of platform governance is in many ways a meta-issue. In recent years, SMCs’ ability to govern themselves has come under questioning, thus launching a broad discourse on the different forms of social media governance. The ethical issues of social media governance are the violation of values, norms and ethical principles of good governance from the various governance perspectives. In this way, the ethical cluster of platform governance is deeply related and involved with each of the ethical issues but is also examined separately as a topic in its own right. In this section we will look in-depth at the three models for governance and the ethical issues of each as suggested in the literature. These ethical issues centre around 1) self-regulation, 2) external governance and 3) third-party governance.

#### Self-Regulation

The ethical issues of self-regulation are the level of responsibility companies are willing to take for ethical externalities versus their self-interest as a business. Self-regulation is a type of minimalist public governance in which responsibility for solving issues falls with the company rather than public regulation (Rochefort, 2020). Most governance in the US is “laissez-faire”, and self-governance has had numerous successes since 2016, such as increasing transparency and requiring political advertisers to register and provide identification (Gorwa, 2019). The advantages of self-regulation are avoiding time-consuming and complicated regulatory procedures and government censorship and reducing cost to the taxpayer (Gorwa, 2019; Rochefort, 2020). However, both Gorwa and Rochefort agree on the limitations of self-regulation: (1) a lack of accountability, (2) no consequences if the company fails to self-regulate, and ultimately (3) being unable to correct the misaligned incentives that lie at the heart of the ABM (Gorwa, 2019; Rochefort, 2020).

#### External Governance

The ethical issues of external governance are the levels of involvement appropriate for centralised governments to have in private companies. External governance, or government regulation, is the most frequently discussed platform governance, which is government involvement in a platform’s governance through regulation. External governance varies from minimal government involvement to comprehensive regulation, or public utilities regulation, where social media platforms are regulated as public goods or public utilities (Rahman, 2018; Rochefort, 2020). Owen (2019) outlines some of the recent regulatory interventions from external governance, such as the European Commission’s report of the independent high-level group on fake news and online disinformation, the UK Parliament’s Digital, Culture, Media and Sport Committee final report, the Knight Commission’s report on trust, media and democracy, the UK government’s online harms white paper, the French government’s report, GDPR and antitrust enforcement. These would be further examples of this type of external governance (Owen, 2019). The risk with this ethical issue is governments controlling the means of communication and information. We will discuss individually the external regulations in more detail in the next section.

#### Third-Party Governance

The ethical issue of third-party governance is the role of semi-state or co-governance bodies addressing the governance issues of social media. Third-party governance or co-governance is suggested as an alternative to the difficulties of individual states being responsible for the international policy of platform regulation (Gorwa, 2019). Some examples of such co-governance in the literature are independent organisations for investigating user complaints to create ethical frameworks for platform companies, ‘ombudsman programs’ and organisations like the Global Network Initiative on freedom of expression and privacy and the EU Internet Forum on the misuse of the internet for terrorist purposes (Gorwa, 2019; Owen, 2019). The majority of records examined in the literature argue that all three forms of governance will be necessary to address the ethical issues of SMCs (Dhawan et al., 2022; Gorwa, 2019; Owen, 2019; Rochefort, 2020) and no one form is sufficient, which is what we will be examining in great detail in the next section.

#### Solutions Proposed

Self-regulation was the default mode for many SMCs but is criticised due to the business model and incentives to maximise engagement (Dhawan et al., 2022). Among the regulatory options suggested are a digital bill of rights (Owen, 2019), harmonisation of regulations of platforms and formal regulations (Winseck, 2022), the development of a higher-order ethical code to ensure technologies (Geeling & Brown, 2021), and “principles-based” regulation (Gorwa, 2019, p. 865). Third-party options between the state and the business themselves have also been suggested (Owen, 2019), alongside more user participation in the platforms, such as voting on policy and design changes (Gorwa, 2019), perhaps resulting in a future of competing and independent platforms (Dhawan et al., 2022) as an alternative to the Web 2 governance structures.

## Discussion

In this section we provide a critical assessment of the state of the literature and insights gleaned from the higher-level perspective arising from this examination of the entire debate. We address the following points: (1) the need for shared concepts and language, (2) The gap between research and regulating the attention economy, and (3) The Need for Mixed Platform Governance

### The Need for Shared Concepts and Language

During the searching stage of the review, it became clear a huge amount of literature is published on social media every year. However, each individual academic field, from medicine to computer science to neuroscience to psychology to law to ethics to economics, approached the topic from within the purview and assumptions of their own discipline. The specialised nature of academic fields and the lack of shared frameworks and methods has hampered not just dealing with the ethical issues of the digital transformation but even agreeing on what these ethical issues are in the first place. As a result, the debate is characterised by multiple overlapping disciplinary analyses of the problems, resulting in semantic and conceptual confusion – the so-called ‘jangle fallacy’.^14^

The word ‘attention’ would be a good example of this problem. The word is employed by every paper dealing with the attention economy, but only a handful define it, and even fewer examine it in detail. Attention is one of the most complex topics in cognitive science and psychology but is addressed in this case by diverse fields such as legal studies, economics, or the social sciences. Most records simply assume an implicit definition of attention that shapes their ethical critique, and without a shared definition (or indeed conception), each record is working with a different framework of the human mind and cognition to understand the harms of social media on users and coming up with contradictory results. The same is true for the term ‘social media’, with some papers explicitly defining social media but most assuming implicit definitions that can be at odds with one another.

To address this confusion, it is important to agree on shared concepts for the analysis of social media and the attention economy. Once a shared theoretical grounding and language are established, the disparate literature can be brought into a levelled account of the various harms across the interdisciplinary work. Much of this work has already been ongoing in the literature in the last ten years, and so there is an opportunity for synthesis across the work done so far. This synthesis would be useful in the development of a lingua franca around emerging technologies or, as William James described, a system of ‘defensible metaphors’ for researchers, policymakers and communication to the general public (Williams, 2017, p. 52).

Academia is part of a larger ecosystem addressing the harms of social media, including regulators, legacy media and indeed the public itself, and in this review we have attempted to provide some shared concepts for these stakeholders across the ethical issues and clusters. During the review we identified several key arenas for developing shared concepts, such as (1) mind, cognition and attention; (2) the relationship between user cognition and technology, for example, ‘cognitive scaffolding’ in the situated cognition literature (Bruineberg, 2023); and (3) the ethical values violated by SMCs, which we have labelled as the ethical clusters, and how these feed into one another and the individual ethical issues.

### The Gap Between Research and Regulating the Attention Economy

In the solutions section, across the literature, it became clear that viable solutions were offered for most of the ethical issues. Many of the ethical issues, such as outrage, misinformation and disinformation (Brady et al., 2020; Rathje et al., 2021); the damaging of trust due to online anonymity, bots, and deep fakes (Geeling & Brown, 2021; Gorwa, 2019; Owen, 2019; Winseck, 2022); and the use of addictive design to hook and manipulate users (Bhargava & Velasquez, 2021; Mujica et al., 2022), are well-documented, and a myriad of solutions have been suggested. However, during the review it became clear that despite viable solutions offered for most of the ethical issues, the academic research was not translating into effective regulation of social media platforms.

The main lever for academic research to effect change on social media platforms is through regulation and policy. While new regulations have come into effect in the EU, i.e., the Digital Markets Act and the Digital Services Act, these focus mostly on content regulation and the companies’ roles as gatekeepers and so have done little to change the incentives and network effects of the attention economy. For example, based on the Digital Markets Act in November 2024, the EU opened a non-compliance investigation on Meta’s new “pay or consent” option for users. This new subscription model was to reduce the collection of users’ data for advertising purposes, but in April 2025, the EU concluded their investigation and fined Meta €200 million for breaching the Digital Markets Act (European Commission, 2025). Meta’s gross revenue in 2024 alone was $164.5B (Meta, 2025), so essentially if Meta can continue to outearn the EU’s fines, then they can ignore the regulations.

Due to this gap between research and regulation in the attention economy, researchers might be better placed to focus on the promotion of digital literacy skills in the population. Based on this review, the most effective digital literacy intervention for the general population would be education on the design of the platforms and how this design is trying to influence their behaviour and perception. While regulation is slow to change the platform, users who are aware of the dark side of social media can actively contribute to a more positive digital environment. In other words, providing digital literacy education on things like the attention economy business model, content-ranking algorithms, gamification and intermittent variable reward schedules helps the general public to gain agency over the platform design while the regulatory battle is ongoing. This literature review and the ethical issues and ethical clusters can be used to inform curricula for digital literacy that focus on equipping knowledge and skills in the general public to reduce harm while the regulatory battle with the platforms is ongoing.

### The Need for Mixed Platform Governance

In this review we have laid out the difficulties of private governance, mainly due to business and economic interests tilting technologies to their own advantage at the cost of users and offline cultures. However, public governments taking control of communication technologies can be equally problematic, as we have seen in authoritarian countries like China and Russia. Government-controlled social media allow states to constantly surveil citizens, create personality models to predict and control behaviour, and use information against political opponents and dissenting voices. This is the key governance issue that emerged during our review: how do democracies steer between the Charybdis of the endlessly predatory profit-maximising behaviour of the private companies and the Scylla of authoritarian control of the communication landscape by the state?

During the review we identified a third way, which we will describe as ‘mixed platform governance’. Mixed platform governance means the platforms remain in control of the private companies, but there is regulatory intervention on the design and systems level. As discussed in the previous section, due to the incentives of the attention economy, the platforms cannot significantly change their design to benefit or at least not harm users, so one solution is that public governments can step in to decouple certain vicious feedback loops that are identified by the academic research. For example, the phenomenon of dis/misinformation and its consequences are not a “bug” in the system of social media but an intrinsic feature of content-ranking algorithms’ behaviour. Therefore, regulation not just on the level of the content itself but rather on how content is distributed on the platform as such could lead to more positive social ends (Shadmy, 2022, p. 3).

In theory, the risks of a government controlling the information landscape are mitigated by allowing targeted interventions on the level of the platforms’ systems and designs rooted in the best research on the downsides of the digital environment. This proposal would be a long-term regulatory solution, in contrast to the “acute” harms usually dealt with by regulation like the DMA and DSA. This type of infrastructural intervention is meant to address the “diffuse chronic” harm of the misaligned internet infrastructure (Harris & Raskin, 2023). A positive rewiring, or ‘reboot’, as was suggested by the inventor of the World Wide Web, Tim Berners-Lee, of the social media environment towards accepted values such as democratic discourse, attentional control, self-awareness, learning, etc:

The goal of regulation is not to achieve an illusory neutrality in social media content moderation. Rather, the goal is to shape the organisation and incentives of the industry to better achieve public ends. (Balkin, 2021, p. 90)

The reason for mixed platform governance, a public redesign of private digital infrastructure, is to correct the negative incentives of the attention economy business model but also avoid the potentially negative incentives of government control of the means of communication. Since the digital infrastructure is increasingly embedded in every level of our social, political, economic and spiritual lives with no signs of retreat, on the longest timescale, a restructuring of the digital infrastructure is inevitable and so should be proactively investigated by researchers to inform policy.

## Conclusion

In this review, we set out to synthesise a complex interdisciplinary literature to chart and reveal connections between the ethical issues attributed to SMCs’ ABM thus far. In undertaking the review, it is clear more interdisciplinary work is needed to bring together the disparate literatures and generate a ‘lingua franca’ for the social media literature. More conceptual work is necessary on the key concepts like attention, but this development has been hampered by a lack of communication between disciplines and shared frameworks. Despite the many viable suggestions for resolving ethical issues in the literature, these are not implemented by the attention industry due to the market incentives. In the meantime, there is a need for education on the platform design and promoting digital literacy about the harms and downsides, which this review can inform. In the long run, an alternative means of mitigating the harms and downsides of the digital environment is necessary, and we have suggested regulation on the design- and systems-level as an alternative to the content regulation paradigm. The possible risks and gains of this type of mixed regulatory intervention are a fruitful field for future researchers to explore.

## Data Availability

This study is based exclusively on published literature. No original data were generated; therefore, a data availability statement is not applicable.

## Appendix

**Search Terms:**

**PsychInfo:**

(“Social Media” OR “Social Network*” OR “Information Technology” OR Facebook OR Instagram OR Snapchat OR “TikTok” OR Youtube OR Twitter) AND (“Attention Engagement” OR “Attention Economy” OR “Attention Monetisation” OR “Attention Economy Business Model” OR “Social Media Business Model”)

### ScienceDirect

(“Social Media” OR “Social Network” OR “Information Technology”) AND (“Attention Engagement” OR “Attention Economy” OR “Attention Monetisation” OR “Social Media Business Model”)

### Scopus & Web of Science

“Social Media” OR “Social Network*” OR “Information Technology” OR Facebook OR Instagram OR Snapchat OR “TikTok” OR Youtube OR Twitter AND “Attention Engagement” OR “Attention Economy” OR “Attention Monetisation” OR “Attention Economy Business Model” OR “Social Media Business Model”

### Google Scholar

“Social Media” OR “Social Network*” OR “Information Technology” OR Facebook OR Instagram OR Snapchat OR TikTok OR Youtube OR Twitter AND “Attention Engagement” OR “Attention Economy” OR “Attention Economy Business Model” OR “Social Media Business Model”

## Footnotes

1 The platforms need to gather these vast audiences to exert “network effects”, bringing more people to the most prominent sites, as, according to Metcalfe’s law, the greater the network, the greater the financial value (Metcalfe, 2013).

2 Records mainly focused on training in marketing, branding, social media management, and becoming an influencer or celebrity were excluded at this point. These records were excluded for not focusing on examining the ethical issues of SMCs with the ABM but rather on how to succeed on the social media platforms and therefore were not relevant to the review.

3 The papers included in autonomy are (Angwald et al., 2021; Aswad, 2020; Atchley & Lane, 2014; Baughan et al., 2022; Beattie, 2022; Bhargava & Velasquez, 2021; Burr & Florida, 2020; Castro & Pham, 2020; Chianella, 2021; Clear, 2022; Fasoli, 2021; Gardiner, 2022; Hanin, 2021; Hartford & Stein, 2021; Jongepier & Klenk, 2022; Kärki, 2022; Karr & Craig, 2019; Kidron et al., 2018; Kozyreva et al., 2020; Lanier, 2018; Lawand, 2021; Lewandowsky et al., 2020; Lukoff et al., 2021; Marin, 2022; McKelvey & Hunt, 2019; Montalvo et al., 2021; Mujica et al., 2022; Paasonen, 2018; Paterson et al., 2021; Pedersen et al., 2021; Pillion et al., 2022; Rufus, 2020; Sahebi & Formosa, 2022; Schoenherr, 2022; Spiekermann et al., 2022; Steinert & Dennis, 2022; Sun & Zhang, 2021; Torbert, 2021; Vanden Abeele et al., 2022 and Williams, 2017).

4 The papers included in manipulation are (Clear, 2022; Fasoli, 2021; Gardiner, 2022; Jongepier & Klenk, 2022; Kärki, 2022; Karr & Craig, 2019; Kidron et al., 2018; Lawand, 2021; Lewandowsky et al., 2020; Lukoff et al., 2021; Marin, 2022; Montalvo et al., 2021; Paasonen, 2018; Paterson et al., 2021; Pedersen et al., 2021; Rufus, 2020; Sahebi & Formosa, 2022; Schoenherr, 2022; Spiekermann et al., 2022; Steinert & Dennis, 2022 and Torbert, 2021).

5 The papers included in Addiction are (Angwald et al., 2021; Aswad, 2020; Baughan et al., 2022; Beattie, 2022; Bhargava & Velasquez, 2021; Burr & Florida, 2020; Castro & Pham, 2020; Chianella, 2021; Hartford & Stein, 2021; Kozyreva et al., 2020; Lanier, 2018; McKelvey & Hunt, 2019; Mujica et al., 2022; Pillion et al., 2022; Sun & Zhang, 2021; Vanden Abeele et al., 2022 and Williams, 2017).

6 The papers included in distraction are (Atchley & Lane, 2014; Hanin, 2021)

7 The papers included in *Unfair Market Practices* are (Baeza-Yates & Fayyad, 2022; Balkin, 2021; Bostoen, 2019; Carah & Brodmerkel, 2022; Cloarec, 2020; Drenten et al., 2020; Goodwin, 2022; Kish, 2020; Linford, 2020; Mittelstadt & Laux, 2021; Myllylahti, 2018; Nelson, 2021; Ormen & Gregersen, 2022; Parisi & Parisi, 2021; Rahman, 2018; Rochefort, 2020; Rosenquist et al., 2022; Wold, 2022; Wu, 2015, 2018 and Zuboff, 2019).

8 The papers included in Privacy & Excessive Advertising are (Baeza-Yates & Fayyad, 2022; Bostoen, 2019; Carah & Brodmerkel, 2022; Cloarec, 2020; Drenten et al., 2020; Goodwin, 2022; Kish, 2020; Linford, 2020; Myllylahti, 2018; Ormen & Gregersen, 2022; Wu, 2015 and Zuboff, 2019).

9 The papers included in attention monopolies are (Balkin, 2021; Mittelstadt & Laux, 2021; Nelson, 2021; Parisi & Parisi, 2021; Rochefort, 2020; Rahman, 2018; Rosenquist et al., 2022; Wold, 2022 and Wu, 2018).

10 The papers included in *Democracy and the Public Sphere* are (Brady et al., 2020; Balsillie, 2019; Buiten, 2022; Collins et al., 2020; Duberry, 2022; Ghosh & Scott, 2018; Heldt & Dreyer, 2021; Hopster, 2021; Kopf, 2022; Pennycook & Rand, 2021; Rauf, 2021; Rathje et al., 2021; Ryan et al., 2019; Scott, 2019; Shadmy, 2022; Stjernfelt & Lauritzen, 2020; Sussman, 2021 and Van Bavel et al., 2021).

11 The papers included in misinformation and disinformation are (Brady et al., 2020; Buiten, 2022; Balsillie, 2019; Ghosh & Scott, 2018; Pennycook & Rand, 2021; Rathje et al., 2021; Ryan et al., 2019; Shadmy, 2022 and Sussman, 2021).

12 The papers included in Content Moderation & Free Speech are (Collins et al., 2020; Duberry, 2022; Heldt & Dreyer, 2021; Kopf, 2022; Scott, 2019 and Stjernfelt & Lauritzen, 2020).

13 The papers included in *Platform Governance* are (Dhawan et al., 2022; Geeling & Brown, 2021; Gorwa, 2019; Owen, 2019; Rochefort, 2020; Scott, 2019 and Winseck, 2022). These selected records are relevant across all three of the three ethical issues.

14 This ‘jangle’ fallacy is that the analyses of different disciplines of the same problems lead to different names and confusion across the interdisciplinary work.

## References

[CR1] Angwald, A., Areskoug, K., & Said, A. (2021). Prediction accuracy and autonomy. *Perspectives on the Evaluation of Recommender Systems Workshop*. 10.48550/arXiv.2211.08134

[CR2] Aswad, E. M. (2020). Losing the freedom to be human. *Columbia Human Rights Law Review*, *52*(1), 307–371. https://search.informit.org/doi/10.3316/agispt.20201204040787

[CR3] Atchley, P., & Lane, S. In B. H. Ross (Ed.). (2014). Chapter 4 - Cognition in the attention economy. In *The psychology of learning and motivation* (pp. 133–177). Elsevier Academic Press. 10.1016/B978-0-12-800283-4.00004-6

[CR4] Baeza-Yates, R., & Fayyad, U. M. (2022). The attention economy and the impact of artificial intelligence. In H. Werthner, E. Prem, E. A. Lee, & C. Ghezzi (Eds.), *Perspectives on digital humanism* (pp. 123–134). Springer. https://doi-org.dcu.idm.oclc.org/10.1007/978-3-030-86144-5_18

[CR5] Balkin, J. M. (2021). How to regulate (and not regulate) social media. *Journal of Free Speech Law*, *71*(2021), 71–96. 10.2139/ssrn.3484114

[CR6] Balsillie, J. (2019). *Six recommendations for the international grand committee on disinformation and “fake news”*. Center for International Governance Innovation. November 16, 2022, from https://www.cigionline.org/articles/six-recommendations-international-grand-committee-disinformation-and-fake-news/

[CR7] Baughan, A., Zhang, M., Rao, R., Lukoff, K., Schaadhardt, A., Butler, D. L., & Hiniker, A. (2022). “I don’t even remember what I read”: How design influences dissociation on social media. In *CHI’22: Proceedings of the 2022 CHI conference on human factors in computing systems*, (Article No: 18), 1–13. https://doi-org.dcu.idm.oclc.org/10.1145/3491102.3501899

[CR8] Beattie, A. (2022). From poacher to protector of attention: The therapeutic turn of persuasive technology and ethics of a smartphone habit-breaking application. *Science, Technology, and Human Values*, *47*(2), 337–359. 10.1177/01622439211042667

[CR9] Bhargava, V. R., & Velasquez, M. (2021). Ethics of the attention economy: The problem of social media addiction. *Business Ethics Quarterly*, *31*(3), 321–359. 10.1017/beq.2020.32

[CR10] Bostoen, F. (2019). Online platforms and pricing: Adapting abuse of dominance assessments to the economic reality of free products. *Computer Law & Security Review*, *35*(3), 263–280. 10.1016/j.clsr.2019.02.004

[CR11] Brady, W. J., Crockett, M. J., & Van Bavel, J. J. (2020). The MAD model of moral contagion: The role of motivation, attention, and design in the spread of moralized content online. *Perspectives on Psychological Science: A Journal of the Association for Psychological Science*, *15*(4), 978–1010. 10.1177/1745691620917336 32511060

[CR12] Bruineberg, J. (2023). Adversarial inference: Predictive minds in the attention economy. *Neuroscience of Consciousness*, *2023*(1), niad 019. 10.1093/nc/niad019 PMC10457025 37635900

[CR13] Buiten, M. (2022). Combating disinformation and ensuring diversity on online platforms: Goals and limits of EU platform. *SSRN Electronic Journal Cyberspace Law eJournal*. 10.2139/ssrn.4009079.

[CR14] Burr, C., & Florida, L. (2020). The ethics of digital well-being: A multidisciplinary perspective. In *Ethics of digital well-being* (pp. 1–29). Springer. 10.1007/978-3-030-50585-1

[CR15] Carah, N., & Brodmerkel, S. (2022). Regulating platforms’ algorithmic brand culture: The instructive case of alcohol marketers on social media. In T. Flew, & F. R. Martin (Eds.), *Digital platform Regulation, (pp. 111-130)*. Palgrave Global Media Policy and Business. Palgrave Macmillan. Springer. https://doi-org.dcu.idm.oclc.org/10.1007/978-3-030-95220-4_6

[CR16] Castro, C., & Pham, A. (2020). Is the attention economy noxious? *Philosophers Imprint*, *20*(17), 1–13.

[CR17] Chianella, R. (2021). Addictive digital experiences: The influence of artificial intelligence and more-than-human design. In *14th International conference of the European Academy of Design, safe harbours for design research* (pp. 414–425). Blucher, São Paulo. 10.5151/ead2021-125

[CR18] Clear, T. (2022). THINKING ISSUES: Algorithms of anger. *ACM Inroads*, *13*(2), 6–8. 10.1145/3530014

[CR19] Cloarec, J. (2020). The personalization–privacy paradox in the attention economy. *Technological Forecasting and Social Change*, *161*, 120299. 10.1016/j.techfore.2020.120299

[CR20] Collins, B., Marichal, J., & Neve, R. (2020). The social media commons: Public sphere, agonism, and algorithmic obligation. *Journal of Information Technology & Politics*, *17*(4), 409–425. 10.1080/19331681.2020.1742266

[CR21] Dhawan, S., Hegelich, S., Sindermann, C., & Montag, C. (2022). Re-start social media, but how? *Telematics and Informatics Reports*, *8*, 100017. 10.1016/j.teler.2022.100017

[CR22] Drenten, J., Gurrieri, L., & Tyler, M. (2020). Sexualized labour in digital culture: Instagram influencers, porn chic and the monetization of attention. *Gender, Work, & Organization*, *27*(1), 41–66. 10.1111/gwao.12354

[CR23] Duberry, J. (2022). AI and the weaponization of information: Hybrid threats against trust between citizens and democratic institutions. In *Artificial intelligence and democracy* (pp. 158–194). Edward Elgar Publishing. Retrieved November 15, 2022 from 10.4337/9781788977319.00011

[CR24] European Commission. (2025, 23rd April). *Commission finds Apple and Meta in breach of the Digital Markets Act* (press release). Retrieved November 1, 2025, from https://ec.europa.eu/commission/presscorner/detail/en/ip_25_1085

[CR25] Fasoli, M. (2021). The overuse of digital technologies: Human weaknesses, design strategies and ethical concerns. *Philosophy & Technology*, *34*(4), 1409–1427. 10.1007/s13347-021-00463-6

[CR26] Gardiner, G. (2022). Attunement: On the cognitive virtues of attention. In M. Alfano, C. Klein, & J. de Ridder (Eds.), *Social virtue epistemology* (pp. 48–72). Routledge.

[CR27] Geeling, S., & Brown, I. (2021). Towards ethical social media practice: A grounded theory for analyzing social media platform ethics. *SSRN Electronic Journal*. 10.2139/ssrn.3942451

[CR28] Ghosh, D., & Scott, B. (2018). The technologies behind precision propaganda on the Internet*. New America, policy papers*. November 16, 2022, from https://www.newamerica.org/pit/policy-papers/digitaldeceit/

[CR29] Goodwin, I. (2022). Programmatic alcohol advertising, social media and public health: Algorithms, automated challenges to regulation, and the failure of public oversight. *International Journal of Drug Policy*, *109*, 103826. 10.1016/j.drugpo.2022.103826 35994939

[CR30] Gorwa, R. (2019). What is platform governance? *Information Communication & Society*, *22*(6), 854–871. 10.1080/1369118X.2019.1573914

[CR31] Hanin, M. L. (2021). Theorizing digital distraction. *Philosophy & Technology*, *34*(2), 395–406. 10.1007/s13347-020-00394-8

[CR32] Harris, T., & Raskin, A. (2023). *The AI dilemma*. The Center for Humane Technology. March 24, 2023, from https://www.humanetech.com/podcast/the-ai-dilemma

[CR33] Hartford, A., & Stein, D. J. (2021). Addiction, autonomy, and the internet: Some ethical considerations. *Mental Health in a Digital World*, 481–495. 10.1016/B978-0-12-822201-0.00003-4

[CR34] Heldt, A., & Dreyer, S. (2021). Competent third parties and content moderation on platforms: Potentials of independent decision-making bodies from a governance structure perspective. *Journal of Information Policy*, *11*, 266–300. 10.5325/jinfopoli.11.2021.0266

[CR35] Hopster, J. (2021). Mutual affordances: The dynamics between social media and populism. *Media Culture & Society*, *43*(3), 551–560. 10.1177/0163443720957889

[CR36] Jongepier, F., & Klenk, M. (2022). Online manipulation: Charting the field. In *The philosophy of online manipulation* (pp. 15–48). Routledge. 10.4324/9781003205425

[CR37] Kärki, K. (2022). Autonomy of attention. In V. C. Müller (Ed.), *Philosophy and theory of artificial intelligence 2021* (pp. 39–55). Springer. 10.1007/978-3-031-09153-7_4

[CR38] Karr, T., & Craig, A. (2019). Beyond fixing Facebook. *Free Press 8, report*. November 16, 2022, from https://www.freepress.net/policy-library/beyond-fixing-facebook

[CR39] Kidron, B., Evans, A., Afia, J., Adler, J., Bowden-Jones, H., Hackett, L., Juj, A., Przybylski, A., Rudkin, A., & Scot, Y. (2018). *Disrupted childhood: The cost of persuasive design* (p. 5Rights). London.

[CR40] Kish, K. (2020). Paying attention: Big data and social advertising as barriers to ecological change. *Sustainability*, *12*(24), 10589. 10.3390/su122410589

[CR41] Kopf, S. (2022). Corporate censorship online: Vagueness and discursive imprecision in YouTube’s advertiser-friendly content guidelines. *New Media and Society*, *26*(4), 1756–1774. https://doi-org.dcu.idm.oclc.org/10.1177/14614448221077354

[CR42] Kozyreva, A., Lewandowsky, S., & Hertwig, R. (2020). Citizens versus the internet: Confronting digital challenges with cognitive tools. *Psychological Science in the Public Interest*, *21*(3), 103–156. 10.1177/1529100620946707 PMC7745618 33325331

[CR43] Lanier, J. (2018). *Ten Arguments for deleting your social media accounts right now*. Random House, Penguin.

[CR44] Lawand, C. (2021). Regulating Facebook: An ethical analysis of AI run amok, political posturing, and the failure of self-regulation. In *University of Ottawa*. *Mémoires // Public and International Affairs*. 10.20381/ruor-26502

[CR45] Lewandowsky, S., Smillie, L., Garcia, D., Hertwig, R., Weatherall, J., Egidy, S., Robertson, R. E., O’Connor, C., Kozyreva, A., Lorenz-Spreen, P., Blaschke, Y., & Leiser, M. (2020). *Technology and democracy: Understanding the influence of online technologies on political behaviour and decision-making*. European Commission. (JRC Science for Policy Report). 10.2760/709177

[CR46] Linford, J. (2020). Copyright and attention scarcity. *Cardozo L. Rev*, *42*, 143.

[CR47] Lukoff, K., Lyngs, U., Zade, H., Liao, J. V., Choi, J., Fan, K., Munson, S. A., & Hiniker, A. (2021). How the design of YouTube influences user sense of agency. In *CHI’21: Proceedings of the 2021 CHI conference on human factors in computing systems.* Article 368 (pp. 1–17). https://doi-org.dcu.idm.oclc.org/10.1145/3411764.3445467

[CR48] Marin, L. (2022). How to do things with information online. A conceptual framework for evaluating social networking platforms as epistemic environments. *Philosophy & Technology*, *35*(3), 1–22. 10.1007/s13347-022-00569-5

[CR49] McDavid, J. (2020). The social dilemma. *Journal of Religion & Film*, *24*(1), Article 22. 10.32873/uno.dc.jrf.24.01.22

[CR50] McKelvey, F., & Hunt, R. (2019). Discoverability: Toward a definition of content discovery through platforms. *Social Media + Society*, *5*(1). 10.1177/2056305118819188

[CR51] Mertz, M., Strech, D., & Kahrass, H. (2017). What methods do reviews of normative ethics literature use for search, selection, analysis, and synthesis? In-depth results from a systematic review of reviews. *Systematic Reviews*, *6*(1), 261. 10.1186/s13643-017-0661-x PMC5738202 29258598

[CR52] Meta. (2025, Jan 29). Meta reports fourth quarter and full year 2024 results. *Meta Investor Relations*. December 10, 2025, from https://investor.atmeta.com/investor-news/press-release-details/2025/Meta-Reports-Fourth-Quarter-and-Full-Year-2024-Results/

[CR53] Metcalfe, B. (2013). Metcalfe’s Law after 40 years of ethernet. *Computer*, *46*(12), 26–31. 10.1109/MC.2013.374

[CR54] Mittelstadt, B., & Laux, J. (2021). Neutralizing online behavioural advertising: Algorithmic targeting with market power as an unfair commercial practice. *Common Market Law Review*, *58*(3), 719–750. 10.54648/cola2021048

[CR55] Montalvo, F. L., Miuccio, M., & Waldfogle, G. (2021). Ethical design of social technology: User engagement and attentional demands. In *Human factors issues and the impact of technology on society, (IGI Global)* (pp. 20–47). 10.4018/978-1-7998-6453-0.ch002

[CR56] Mujica, A. L., Crowell, C., Villano, M., & Uddin, K. (2022). Addiction by design: Some dimensions and challenges of excessive social media use. *Medical Research Archives*, *10*(2). 10.18103/mra.v10i2.2677

[CR57] Myllylahti, M. (2018). An attention economy trap? An empirical investigation into four news companies’ Facebook traffic and social media revenue. *Journal of Media Business Studies*, *15*(4), 237–253. 10.1080/16522354.2018.1527521

[CR58] Nelson, J. (2021). Tech platforms are essential facilities. *Nevada Law Journal*, *22*(1), 10

[CR59] Ormen, J., & Gregersen, A. (2022). Towards the engagement economy: Interconnected processes of commodification on YouTube. *Media Culture & Society*, *45*(2), 225–245. 10.1177/01634437221111951

[CR60] Owen, T. (2019). The case for platform governance. Centre for International Governance Innovation. November 16, 2022, from https://www.cigionline.org/publications/case-platform-governance

[CR61] Paasonen, S. (2018). Affect, data, manipulation and price in social media. *Distinktion: Journal of Social Theory*, *19*(2), 214–229. 10.1080/1600910X.2018.1475289

[CR62] Parisi, E. C., & Parisi, F. (2021). Rethinking remedies for the attention economy. In *Research in law and economics, in:* The economics and regulation of digital markets (Vol. 31, pp. 75–97). Emerald Group Publishing Limited. 10.1108/S0193-589520240000031004

[CR63] Paterson, J. M., Chang, S., Cheong, M., Culnane, C., Dreyfus, S., & McKay, D. (2021). The hidden harms of targeted advertising by algorithms and interventions from the consumer protection toolkit. *SSRN Electronic Journal*, *9*. 10.2139/ssrn.3993496

[CR64] Pedersen, M. A., Albris, K., & Seaver, N. (2021). The political economy of attention. *Annual Review of Anthropology*, *50*(1), 309–325. 10.1146/annurev-anthro-101819-110356

[CR65] Pennycook, G., & Rand, D. G. (2021). The psychology of fake news. *Trends in Cognitive Sciences*, *25*(5), 388–402. 10.1016/j.tics.2021.02.007 33736957

[CR66] Pillion, M., Gradisar, M., Bartel, K., Whittall, H., & Kahn, M. (2022). What’s “app”-ning to adolescent sleep? Links between device, app use, and sleep outcomes. *Sleep Medicine*, *100*, 174–182. 10.1016/j.sleep.2022.08.004 36084495

[CR67] Rahman, K. S. (2018). Regulating informational infrastructure: Internet platforms as the new public utilities. *Georgetown Law and Technology Review*, *2*, 2.

[CR68] Rathje, S., Van Bavel, J. J., & Van der Linden, S. (2021). Out-group animosity drives engagement on social media. *Proceedings of the National Academy of Sciences*, *118*(26), e2024292118. 10.1073/pnas.2024292118 PMC8256037 34162706

[CR69] Rauf, A. A. (2021). New moralities for new media? Assessing the role of social media in acts of terror and providing points of deliberation for business ethics. *Journal of Business Ethics*, *170*(2), 229–251. 10.1007/s10551-020-04635-w. https://doi-org.dcu.idm.oclc.org/10.1007/s10551-020-04635-w

[CR70] Rochefort, A. (2020). Regulating social media platforms: A comparative policy analysis. *Communication Law and Policy*, *25*(2), 225–260. 10.1080/10811680.2020.1735194

[CR71] Rosenquist, J., Scott Morton, F., & Weinstein, S. (2022). Addictive technology and its implications for antitrust enforcement. *North Carolina Law Review*, *100*, 431–486. 10.2139/ssrn.3787822

[CR72] Rufus, H. (2020). The implications of personalisation algorithms on individual autonomy. *SACAIR*, *36*.

[CR73] Ryan, C. D., Schaul, A., Butner, R., & Swarthout, J. (2019). Monetizing disinformation in the attention economy: The case of genetically modified organisms (GMOs). *European Management Journal* (Special Issue: “The Dark Side of Social Media”). 10.1016/j.emj.2019.11.002

[CR74] Sahebi, S., & Formosa, P. (2022). Social media and its negative impacts on autonomy. *Philosophy & Technology*, *35*(3), 1–24. 10.1007/s13347-022-00567-7

[CR75] Schoenherr, J. (2022). The currency of the attentional economy: The uses and abuses of attention in our world. *IEEE Technology and Society Magazine*, *41*(1), 11–14. 10.1109/MTS.2022.3147541

[CR76] Scott, T. (2019). *Facing down Facebook: Reclaiming democracy in the age of (anti) social media*. Falmouth University Research Repository. November 16, 2022, from http://www.mollymep.org.uk/

[CR77] Shadmy, T. (2022). Content traffic regulation: A democratic framework for addressing misinformation. *Jurimetrics J*, *63*, 1–57.

[CR78] Spiekermann, S., Krasnova, H., Hinz, O., Baumann, A., Benlian, A., Gimpel, H., Heimbach, I., Köster, A., Maedche, A., Niehaves, B., Risius, M., & Trenz, M. (2022). Values and ethics in information systems. *Business & Information Systems Engineering*, *64*(2), 247–264. 10.1007/s12599-021-00734-8

[CR79] Steinert, S., & Dennis, M. J. (2022). Emotions and digital well-being: On social media’s emotional affordances. *Philosophy & Technology*, *35*(2), 1–21. 10.1007/s13347-022-00530-6 PMC9007765 35450167

[CR80] Stjernfelt, F., & Lauritzen, A. M. (2020). *Your post has been removed: Tech giants and freedom of speech*. Springer Nature.

[CR81] Sun, Y., & Zhang, Y. (2021). A review of theories and models applied in studies of social media addiction and implications for future research. *Addictive Behaviors*, *114*, 106699. 10.1016/j.addbeh.2020.106699 33268185

[CR82] Sussman, K. (2021). Detecting effects of misinformation through emotion and trace behavior. In *TPRC49: The 49th research conference on communication*, *information and internet policy*. 10.2139/ssrn.3911984

[CR88] The Wall Street Journal. (2021). *The Facebook files*. December 20, 2023, from https://www.wsj.com/articles/the-facebook-files-11631713039

[CR83] Torbert, P. M. (2021). Because it is wrong: The immorality and illegality of the online service contracts of Google and Facebook. *SSRN Electronic Journal*, *12*(1), 2. 10.2139/ssrn.3537227

[CR84] Tricco, A. C., Lillie, E., Zarin, W., O’Brien, K. K., Colquhoun, H., Levac, D., Moher, D., Peters, M. D., Horsley, T., Weeks, L., Hempel, S., Akl, E. A., Chang, C., McGowan, J., Stewart, L., Hartling, L., Aldcroft, A., Wilson, M. G.Garritty, C. … Straus, S. E. (2018). PRISMA extension for scoping reviews (PRISMA-ScR): Checklist and explanation. *Annals of Internal Medicine*, *169*(7), 467–473. 10.7326/M18-0850 30178033

[CR85] Vallor, S. (2022). Social networking and ethics. In N. Z. Edward, & U. Nodelman (Eds.), *The stanford encyclopedia of philosophy*. October 30, 2023. (Fall Edition). https://https://plato.stanford.edu/archives/fall2022/entries/ethics-social-networking

[CR86] Van Bavel, J. J., Rathje, S., Harris, E., Robertson, C., & Sternisko, A. (2021). How social media shapes polarization. *Trends in Cognitive Sciences*, *25*(11), 913–916. 10.1016/j.tics.2021.07.013 34429255

[CR87] Vanden Abeele, M. M. P., Halfmann, A., & Lee, E. W. (2022). Drug, demon, or donut? Theorizing the relationship between social media use, digital well-being and digital disconnection. *Current Opinion in Psychology*, *45*, 45. 10.1016/j.copsyc.2021.12.007 35123383

[CR89] Williams, J. (2017). *Freedom and persuasion in the attention economy* [Doctoral dissertation]. University of Oxford. Oxford University Research Archive, available at: https://ora.ox.ac.uk/objects/uuid:8065d7b0-4125-4b1f-9218-8623380e03d7

[CR90] Winseck, D. (2022). The broken internet and platform regulation: Promises and perils. In T. Flew, *&* F. R. Martin (Eds.), *Digital platform regulation* (pp. 229–257). Springer. https://doi-org.dcu.idm.oclc.org/10.1007/978-3-030-95220-4_12

[CR91] Wold, T. L. (2022). Not a foregone conclusion: The early history of Facebook’s political economy of social media. *Artifact Analysis*, *1*(1).

[CR92] Wu, T. (2018). Blind spot: The attention economy and the law. *Antitrust LJ*, *82*, 771–801. 10.2139/ssrn.2941094

[CR93] Wu, T. (2015). *Attention brokers*. November 18, 2022, https://www.law.nyu.edu/sites/default/files/upload_documents/Tim%20Wu%20-%20Attention%20Brokers.pdf

[CR94] Zuboff, S. (2019). *The age of surveillance capitalism*. Profile Books.

[CR95] Zuckerman, E. (2014, August 14). The internet’s original sin. *The Atlantic*. April 29, 2025, from https://www.theatlantic.com/technology/archive/2014/08/advertising-is-the-internets-original-sin/376041/

